# Solvent-Free Synthesis of Ultrafine Tungsten Carbide Nanoparticles-Decorated Carbon Nanosheets for Microwave Absorption

**DOI:** 10.1007/s40820-020-00491-5

**Published:** 2020-07-18

**Authors:** Yunlong Lian, Binhua Han, Dawei Liu, Yahui Wang, Honghong Zhao, Ping Xu, Xijiang Han, Yunchen Du

**Affiliations:** MIIT Key Laboratory of Critical Materials Technology for New Energy Conversion and Storage, School of Chemistry and Chemical Engineering, Harbin Institute of Technology, Harbin, 150001 People’s Republic of China

**Keywords:** Solvent-free synthesis, Tungsten carbide/carbon composite, Ultrafine nanoparticle, Microwave absorption, Dielectric loss

## Abstract

Carbides/carbon composites are emerging as a new kind of binary dielectric systems with good microwave absorption performance. Herein, we obtain a series of tungsten carbide/carbon composites through a simple solvent-free strategy, where the solid mixture of dicyandiamide (DCA) and ammonium metatungstate (AM) is employed as the precursor. Ultrafine cubic WC_1−*x*_ nanoparticles (3–4 nm) are in situ generated and uniformly dispersed on carbon nanosheets. This configuration overcomes some disadvantages of conventional carbides/carbon composites and is greatly helpful for electromagnetic dissipation. It is found that the weight ratio of DCA to AM can regulate chemical composition of these composites, while less impact on the average size of WC_1−*x*_ nanoparticles. With the increase in carbon nanosheets, the relative complex permittivity and dielectric loss ability are constantly enhanced through conductive loss and polarization relaxation. The different dielectric properties endow these composites with distinguishable attenuation ability and impedance matching. When DCA/AM weight ratio is 6.0, the optimized composite can produce good microwave absorption performance, whose strongest reflection loss intensity reaches up to − 55.6 dB at 17.5 GHz and qualified absorption bandwidth covers 3.6–18.0 GHz by manipulating the thickness from 1.0 to 5.0 mm. Such a performance is superior to many conventional carbides/carbon composites.

**Electronic supplementary material:**

The online version of this article (10.1007/s40820-020-00491-5) contains supplementary material, which is available to authorized users.

## Highlights

- Ultrafine tungsten carbide nanoparticles-decorated carbon nanosheets were successfully fabricated via a simple solvent-free strategy.
- The chemical composition of tungsten carbide/carbon composites can be easily manipulated by the weight ratio of dicyandiamide to ammonium metatungstate.
- The advantages in good performance and simple preparation provide a promising prospect for the application of these tungsten carbide/carbon composites.

**Electronic supplementary material:**

The online version of this article (10.1007/s40820-020-00491-5) contains supplementary material, which is available to authorized users.

## Graphical abstract

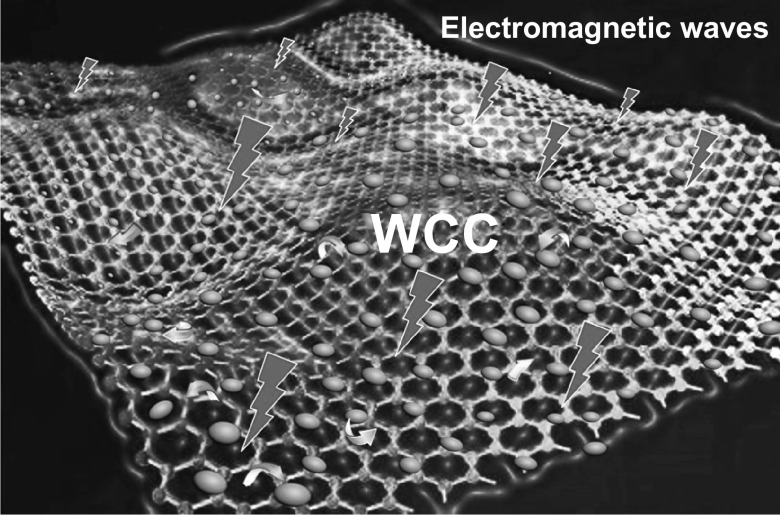

## Introduction

Electromagnetic (EM) pollution has become a global concern owing to its potential threats in physical health, equipment operation, and information security [1–4]. Although the shielding strategy can produce powerful effectiveness in individual protection, the reflection principle therein makes it invalid to alleviate those side effects of EM technology [5, 6]. In the past two decades, microwave absorption emerged as an advanced and sustainable alternative for the precaution of EM pollution, because this promising strategy was established on the fundamental energy conversion through the interaction between EM waves and microwave-absorbing materials (MAMs) [7, 8]. Among various functional materials with unique EM characteristics, carbon materials are receiving more and more attention due to their desirable advantages in tunable dielectric property, low density, chemical inertness, and designable microstructure [9–11]. It is unfortunate that single-component carbonaceous MAMs easily suffer from mismatched impedance with that of free space, which will result in their poor microwave absorption performance as most of EM waves are reflected off at the interface rather than being transmitted into MAMs [3, 12]. In an effort to reinforce the consumption of EM energy, there are numerous efforts that have been devoted to carbon-based composites by introducing some magnetic components (i.e., magnetic metals and ferrites) [13–17], while the intrinsic drawbacks of magnetic components in carbon matrix, including easy corrosion, weak magnetic loss, and Curie temperature limitation, restrain the practical application of these composites to some extent [18–20]. As a result, many researchers started to focus on the fabrication of non-magnetic systems that combine carbon materials and secondary dielectric components [21–23]. Our group recently designed core–shell BaTiO_3_@C microspheres as a binary dielectric system, and we found that it displayed comparable microwave absorption performance but superior corrosion resistance to those common magnetic carbon-based composites [24].

Carbides, as a kind of special ceramics, have good mechanical, physical, and chemical properties that make them active in many fields ranging from energy storage to catalysis [25, 26]. In particular, their intrinsic characteristics of polarization relaxation also render them as popular dielectric components to couple with carbon materials for EM attenuation [27, 28]. A lot of successful examples have demonstrated the positive synergistic effects between carbon and carbides that could enhance the microwave absorption performance of these composites effectively [29–31]. However, in most cases, the selection of carbides was usually limited to SiC or Ti_3_C_2_ Mxenes. It is well known that either SiC or Ti_3_C_2_ has very large particle size even up to several microns, which means that these carbides particles cannot fully contact with carbon matrix, and thus, the contribution from interfacial polarization may be insufficient in such carbon/carbide composites [29, 32]. More recently, some attempts were made to decorate carbon matrix with some unconventional carbide particles, whose ultrafine particle size could generate good dispersion and abundant interfaces, resulting in a significant enhancement in microwave absorption [33–37]. For example, Dai et al. pioneered the involvement of Mo_2_C nanoparticles in carbon matrix through the pyrolysis of Cu-Mo-based metal organic frameworks (MOFs) and the subsequent removal of Cu species, and they confirmed that the synergistic effect of carbon frameworks and Mo_2_C nanoparticles were responsible for the good reflection loss (RL) characteristics [34]. Li et al. conducted the coating of carbonaceous layer on pre-prepared MoO_3_ nanowires through hydrothermal carbonization of glucose solution and then transformed this precursor into final Mo_2_C@C nanowires under a high-temperature inert atmosphere, and they demonstrated that the composition and microstructure were favorable for the consumption of EM energy by multiple polarization relaxations [35]. In our previous work, we also harvested Mo_2_C/C composites from Mo-substituted ZIF-8, whose microwave absorption performance was found to be superior to those counterparts with SiC and Ti_3_C_2_ [37]. Although some positive achievements have been identified in these carbon-based composites with ultrafine carbide particles, it is worth noting that the formation of such composites is always complex and time-consuming. Therefore, a simple method is highly desirable to produce carbon-based composites with uniform dispersion of ultrafine carbide particles.

Herein, we report a solvent-free route for tungsten carbide/carbon composites, which only requires grinding the mixture of dicyandiamide (DCA, carbon source) and ammonium metatungstate (AM, tungstate source) before high-temperature pyrolysis. The final products are composed of highly dispersed ultrafine tungsten carbide nanoparticles (ca. 3–4 nm) and amorphous carbon matrix, and their EM properties and RL characteristics can be easily regulated by the weight ratio of DCA to AM. When the composition is optimized, the resultant tungsten carbide/carbon can produce good microwave absorption with the strongest RL intensity of − 55.6 dB and the effective absorption bandwidth of 14.4 GHz (3.6–18.0 GHz, 1.0–5.0 mm). Compared with those common wet chemical ways, this solvent-free strategy is obviously advantageous of simple operation, saving time, environmental benign, and scale-up production [38, 39], and thus, this study may open a new avenue for the fabrication of carbide/carbon composites.

## Experimental Section

### Synthesis

**Fig. 1:**
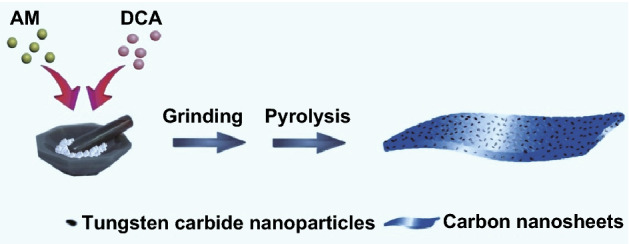
Schematic illustration for solvent-free synthesis of different tungsten carbide/carbon composites

A typical procedure for tungsten carbide/carbon composite is illustrated in Fig. 1. First, 0.5 g of AM was added into an agate mortar, and then, a required amount of DCA was also introduced. The mixture was sufficiently ground for 15 min. After that, the mixture was transferred into a porcelain boat and pyrolyzed in a horizontally tubular furnace under N_2_ nitrogen at 400 °C for 0.5 h and 800 °C for 5 h, respectively. The heating rates from room temperature to 400 °C and from 400 to 800 °C were 2 and 5 °C min^−1^, respectively. The final products were denoted as WCC-*r*, where *r* referred to the weight ratio of DCA to AM.

### Characterization

Powder X-ray diffraction (XRD) data were recorded on a Rigaku D/MAXRC X-ray diffractometer (45.0 kV, 50.0 mA) using Cu Kα source. Raman spectra were recorded on a confocal Raman spectroscopic system (Renishaw, In Via) using a 633 nm laser. X-ray photoelectron spectroscopy (XPS, Kratos, ULTRA AXIS DLD) was recorded to study the surface states with monochrome Al Kα (1486.6 eV) radiation. Transmission electron microscopy (TEM) images were obtained on a Tecnai F20 instrument operating at an accelerating voltage of 200 kV. The thermogravimetric analysis was carried out on a SDT Q600 thermogravimetric analyzer (TGA) in the temperature range of room temperature to 800 °C at a heating rate of 10 °C min^−1^ under air atmosphere. The conductivities of WCC-*r* were recorded on a four-probe resistivity meter (RTS-9, Guangzhou 4-probes Technology Co., Ltd, China). Before the measurement, 45 wt% of WCC-*r* and 55 wt% of molten paraffin wax were adequately ground for about 30 min to obtain a uniform mixture, and then, the mixture was collected in a metallic disk with a diameter of 20 mm and a thickness of 0.30 mm for pressing into a circular sheet through a tablet machine. The relative complex permittivity and complex permeability in the frequency range of 2.0–18.0 GHz were measured using an Agilent N5234A vector network analyzer (Agilent, USA) for the calculation of reflection loss characteristics. A sample containing 45 wt% of obtained composites was pressed into a ring with an outer diameter of 7.0 mm, an inner diameter of 3.0 mm, and a thickness of 2.0 mm for microwave measurement in which paraffin wax was used as the binder. The frequency span during the measurement was 0.08 GHz, and the symbols in the related curves were labeled at a given interval for clarification.

## Results and Discussion

### Structure Characterization

**Fig. 2:**
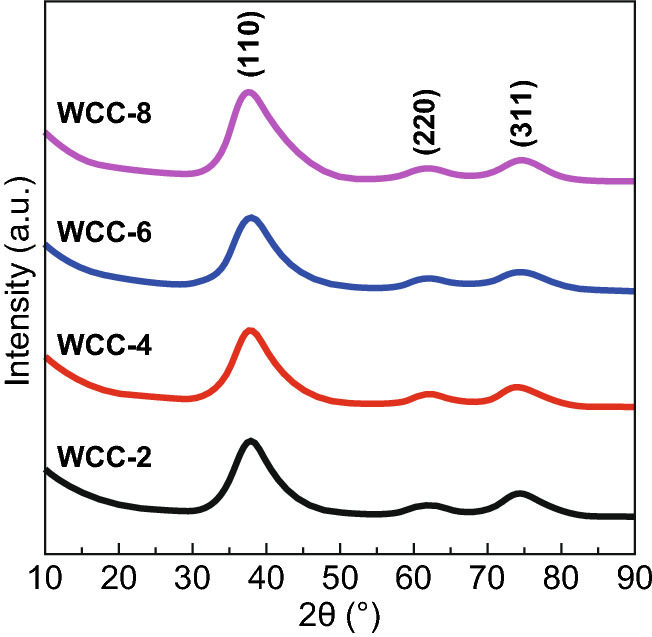
XRD patterns of different tungsten carbide/carbon composites

Figure 2 shows XRD patterns of tungsten carbide/carbon composites from different DCA/AM weight ratios. It can be seen that all samples exhibit three identical and well-resolved peaks at 37.0°, 62.0°, and 74.2°. Tungsten carbide has long been known to have three crystalline structures, namely hexagonal WC phase, close-packed hexagonal W_2_C phase, and face-centered cubic WC_1−*x*_ phase [40]. The three peaks of these composites can be precisely matched with (111), (220), and (311) planes of WC_1−*x*_ [41, 42], respectively, indicating that tungsten carbide mainly exists as WC_1−*x*_ phase in these composites. The obvious broadening of these peaks means that WC_1−*x*_ in these composites has very small particle size. With these measured patterns, one can calculate that the average cell parameter (*a*) of cubic WC_1−*x*_ nanoparticles herein is about 0.4223 nm. Generally speaking, WC_1−*x*_ phase is usually generated under a carbon-rich condition [43]. Although the minimum weight ratio of DCA to AM is only 2.0, the real C/W molar ratio exceeds 10.0, and thus, the formation of WC_1−*x*_ nanoparticles in these composites is understandable. Of note is that a larger dosage of DCA will not impact the crystalline phase of tungsten carbide nanoparticles any more. The large excess of DCA not only facilitates the formation of WC_1−*x*_ nanoparticles, but also produces some individual carbon components, while these individual carbon components fail to present any characteristic peaks due to their amorphous nature. Kurlov and Gusev ever investigated the phase transformation of tungsten carbide particles in detail [44], and they proposed that *a* was a parabolic function of carbon content in WC_1−*x*_ particles, and their relationship could be described by Eq. 1.

1 $$ a = 0.4015 + 0.0481(1 - x) - 0.0236(1 - x)^{2}. $$

Based on Eq. 1, we get the value of 1 − *x* is about 0.62 using 0.4223, and thus, the specific expression of tungsten carbide nanoparticles in these composites should be WC_0.62_. Moreover, the survey of XPS spectra also detects very weak signals of N species at about 400 eV (Fig. S1), suggesting that some N atoms in DCA are preserved in those individual carbon components. These N species can be considered as defective sites in carbon components, and thus, they can produce positive effect on the consumption of EM energy through polarization loss [11].

**Fig. 3:**
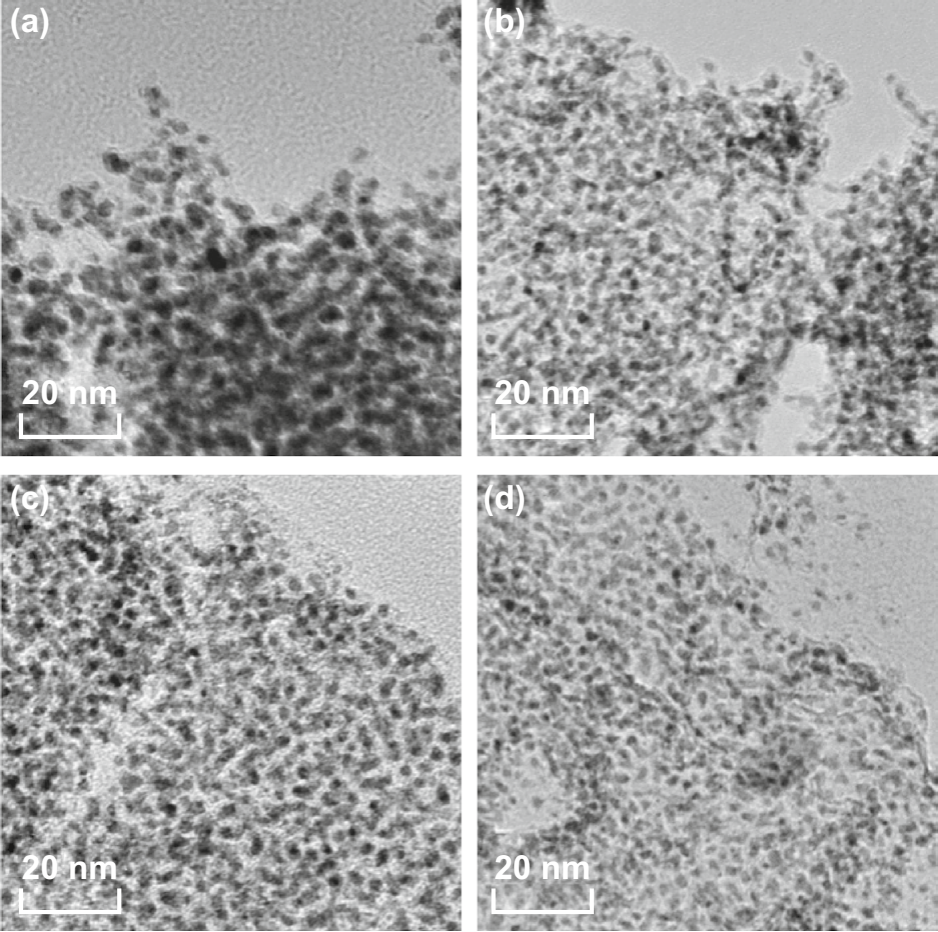
TEM images of **a** WCC-2, **b** WCC-4, **c** WCC-6, and **d** WCC-8

In order to better understand the microstructure of these tungsten carbide/carbon composites, TEM measurement is further carried out. As shown in Fig. S2, one can see that numerous ultrafine WC_1−*x*_ nanoparticles are uniformly dispersed on carbon nanosheets. It is believable that such good dispersion of WC_1−*x*_ nanoparticles on carbon nanosheets will create sufficient interfaces and be greatly helpful for the consumption of EM energy [45, 46]. With the increase in DCA/AM weight ratios, the deposition density of WC_1−*x*_ nanoparticles on carbon nanosheets is gradually decreased from WCC-2 to WCC-8, indicating that the chemical composition of these composites can be easily manipulated according to the dosages of AM and DCA. A closer inspection reveals that the average sizes of WC_1−*x*_ nanoparticles in these composites are all ca. 3–4 nm (Fig. 3). That is to say, the weight ratio of DCA to AM just regulates the relative contents of WC_1−*x*_ nanoparticles and carbon nanosheets rather than the crystalline structure and size of WC_1−*x*_ nanoparticles. In view of the fact that solvent-free synthesis has extremely high utilization efficiency of metal atoms and does not produce any wastewater, it is an absolutely green and sustainable strategy for tungsten carbide/carbon composites.

**Fig. 4:**
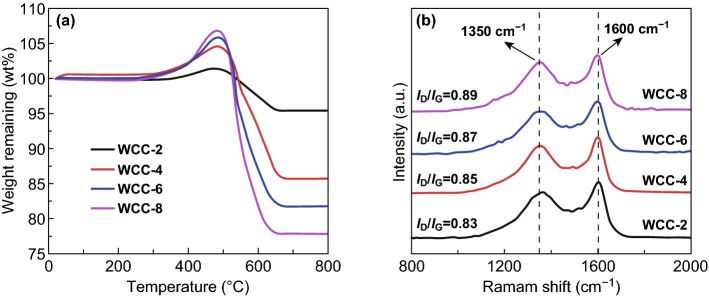
**a** TG curves and **b** Raman spectra of different tungsten carbide/carbon composites

It is well known that EM properties of carbon-based composites are highly dependent on the relative content and graphitization degree of carbon components, especially for those without magnetic components [24, 47]. TG is an effective characterization method to determine carbon content in carbon-based composites, because carbon species will be totally removed at high temperature under air atmosphere [17, 48]. As shown in Fig. 4a, all four composites display quite similar TG curves that contain a weight increase region and a weight loss region. The former is attributed to the oxidation of WC_1-*x*_ nanoparticles, and the latter should be resulted from the combustion of carbon nanosheets. We characterize the residual powder after TG measurement and confirm that these composites will be completely transformed into WO_3_ (Fig. S3). In other words, not only carbon nanosheets but also carbon atoms in WC_1−*x*_ nanoparticles will be also removed simultaneously. Based on the possible expression of WC_1−*x*_ nanoparticles by XRD patterns, one can estimate the specific content of carbon nanosheets with Eq. 2:

2 $$ R\;{\text{wt}}\% = (1 - C\;{\text{wt}}\% )\frac{{M_{{{\text{WO}}_{3} }} }}{{M_{{{\text{WC}}_{1 - x} }} }} $$

where *R* wt%, C wt%, $M_{{{\text{WO}}_{3} }}$, and $M_{{{\text{WC}}_{1 - x} }}$ are referred to remaining weight percentage, carbon nanosheets content, WO_3_ formula weight, and WC_1−*x*_ formula weight, respectively. The calculation results suggest that the specific contents of carbon nanosheets in WCC-2, WCC-4, WCC-6, and WCC-8 are 21.3%, 29.3%, 32.6%, and 35.8%, respectively, which mean that the chemical composition of these composites can be finely regulated by the weight ratio of DCA to AM. However, due to the gap in formula weights between C and WC_1-*x*_, the large span of weight ratio from 2.0 to 8.0 only accounts the increase in the relative content of carbon nanosheets from 21.3 to 35.8%. In addition, these composites give quite similar onset temperature (ca. 300 °C) for weight increase, again verifying that the average sizes of WC_1−*x*_ nanoparticles in different composites are much pretty close, as indicated by TEM images (Fig. 2). WCC-8 has the lowest content of WC_1-*x*_ nanoparticles, while it promises the highest weight increase at about 480 °C. Obviously, the good dispersion of WC_1−*x*_ nanoparticles in WCC-8 accounts for an intensive oxidation.

Figure 4b shows Raman spectra of tungsten carbide/carbon composites from different DCA/AM weight ratios. As observed, all of them present two distinguishable bands at 1350 and 1600 cm^−1^, which are typical bands in carbon components and widely denoted as D band and G band, respectively [47, 49]. Ferrari and Robertson ever interpreted the evolution in Raman spectra from amorphous carbon to perfect graphite in detail [49]. They proposed that the change in the bonding state of carbon atoms could be linked with three features, G band position, G band profile, and the intensity ratio of D band to G band (*I*_D_/*I*_G_). During the stage from amorphous carbon to nanocrystalline graphite, the position of G band gradually shifts from 1510 to 1600 cm^−1^ and the profile of G band becomes relatively narrow, and meanwhile, an incremental *I*_D_/*I*_G_ value from 0.25 to 2.0 can be also detected. These changes are attributed to the decreased disorders in bond angle and bond bending, as well as the tightened vibrational density of states. However, these tiny crystalline domains are very difficult to detect, even by high-resolution TEM [50, 51]. When nanocrystalline graphite is further transformed into perfect graphite, all these changes will be inverse. In our case, the positions of these tungsten carbide/carbon composites are all located at 1600 cm^−1^, while the local amplification reveals that the values of full-width half-maximum in G band for WCC-2, WCC-4, WCC-6, and WCC-8 are 110, 106, 96, and 88 cm^−1^, respectively (Fig. S4). At the same time, one can also find that *I*_D_/*I*_G_ values slowly increase from 0.83 to 0.89. These phenomena suggest the formation of nanocrystalline graphite domains in amorphous carbon nanosheets, that is, more W species may decrease the relative graphitization degree of carbon nanosheets to some extent.

### Dielectric Property

**Fig. 5:**
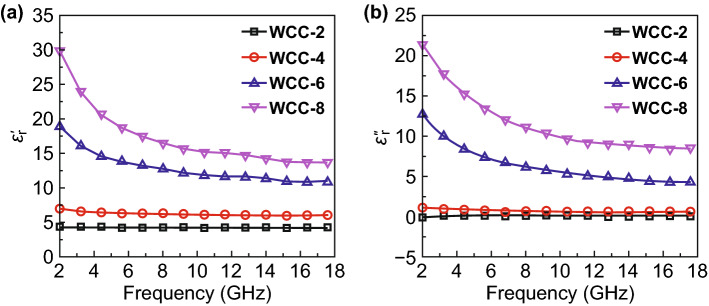
**a** Real parts and **b** imaginary parts of relative complex permittivity of different tungsten carbide/carbon composites

Relative complex permittivity (*ε*_r_ = *ε*_r_′ − j*ε*_r_″) and complex permeability (*μ*_r_ = *μ*_r_′ − j*μ*_r_″) are two extremely important parameters to determine the performance of MAMs [17, 52]. Herein, all tungsten carbide/carbon composites are free of any magnetic components, and thus, their real and imaginary parts of complex permeability are constant at 1 and 0, respectively (Fig. S5). That is to say, dielectric loss is the only pathway to attenuate incident EM energy [24, 36]. Figure 5 shows *ε*_r_′ and *ε*_r_″ curves of different tungsten carbide/carbon composites in the frequency range of 2.0–18.0 GHz. Among them, WCC-2 has the lowest *ε*_r_′ and *ε*_r_″ values, which are insusceptible to the frequency and almost constant at 4.35 and 0.24, respectively. When more carbon nanosheets are introduced, both *ε*_r_′ and *ε*_r_″ values are significantly increased. Especially for WCC-6 and WCC-8, they not only have larger *ε*_r_′ and *ε*_r_″ values, but also present typical frequency dispersion behaviors. For example, *ε*_r_′ and *ε*_r_″ values of WCC-6 gradually decrease from 18.87 and 12.60 at 2.0 GHz to 10.87 and 4.25 at 18.0 GHz, respectively, and *ε*_r_′ and *ε*_r_″ values of WCC-8 gradually decrease from 29.84 and 21.09 at 2.0 GHz to 13.33 and 8.43 at 18.0 GHz, respectively. Dielectric loss tangent (tan*δ*_e_ = *ε*_r_″/*ε*_r_′) further demonstrates that the dielectric loss ability of these composites is continuously enhanced with the increase in carbon nanosheets (Fig. S6).

It is believable that this enhancement should be associated with the following two aspects: conductive loss and polarization loss [53–55]. Conductive loss is always generated by some residual carriers in dielectric medium, and their directional movement under an applied electric field will convert electric energy into heat energy [56]. In general, carbon component has better conductivity than WC_1−*x*_ nanoparticles [57], and thus, the increase in carbon nanosheets can improve the conductivity of tungsten carbide/carbon composites. Four-probe measurement also reveals that the conductivities of WCC-2, WWC-4, WCC-6, and WCC-8 in wax are 4.21 × 10^−5^, 2.03 × 10^−4^, 8.71 × 10^−4^, and 5.29 × 10^−3^ S m^−1^, respectively. Although the relative content of carbon nanosheets is not remarkably increased, its lower density as compared with WC_1-*x*_ nanoparticles may promise a larger volume fraction, resulting in the formation of conductive networks in wax matrix and considerable contribution to conductive loss.

Compared to conductive loss, polarization loss has diversified modes, i.e., electronic polarization, ionic polarization, dipole orientation polarization, and interfacial polarization [54, 55]. However, electronic polarization and ionic polarization are widely accepted to be inactive for the dissipation of EM energy in gigahertz range because their relaxation time is too short (10^−12^–10^−16^ s) [54, 55]. Therefore, dipole orientation polarization and interfacial polarization are the two modes that can be responsible for energy consumption under current conditions. Dipole orientation polarization usually comes from the hysteretic reorientation of dipoles along with an applied electric field [58, 59]. In our case, there are two kinds of dipole orientation polarization relaxations. One is from intrinsic dipoles in WC_1-*x*_ nanoparticles, and the other is from bound charges in residual functional groups and defective sites (N sites) in carbon nanosheets. With the increase in carbon nanosheets, the number of intrinsic dipoles in WC_1−*x*_ nanoparticles will decrease, while bound charges in carbon nanosheets may make up the loss of dipoles, and thus, the contribution of dipole orientation polarization will not significantly change.

As for interfacial polarization, it mainly depends on the asymmetrical accumulation of space charges at heterogeneous interfaces, which can generate an electric dipole moment to drive energy consumption [45, 54]. That is to say, more heterogeneous interfaces can produce more powerful interfacial polarization. The increase in carbon nanosheets alleviates the aggregation of WC_1−*x*_ nanoparticles effectively (Fig. S2), and thus, there will be more interfaces between WC_1−*x*_ nanoparticles and carbon nanosheets from WCC-2 to WCC-8, accounting for the enhancement of interfacial polarization. Actually, when polarization loss works for the dissipation of EM energy, frequency dispersion behaviors may occur in *ε*_r_′ and *ε*_r_″ curves [36, 58], as indicated in Fig. 5. Meanwhile, the contribution of polarization loss can also be witnessed by several semicircles in the curves of *ε*_r_″ versus *ε*_r_′ (Fig. S7), where each semicircle (denoted as Cole–Cole semicircle) corresponds to one polarization relaxation according to Debye theory [47, 55]. However, Cole–Cole semicircles just describe the number of relaxation processes, but not their intensities. The less semicircles in WCC-6 and WCC-8 may be attributed to the fact that the decrease in the relative content of WC_1−*x*_ nanoparticles weakens their intrinsic dipole orientation polarization. From the above analyses, it can be concluded that the overall dielectric loss is the sum of conductive loss, dipole orientation polarization, and interfacial polarization, while conductive loss and interfacial polarization play two more progressive roles to reinforce dielectric loss from WCC-2 to WCC-8.

### Microwave Absorption and Mechanism

**Fig. 6:**
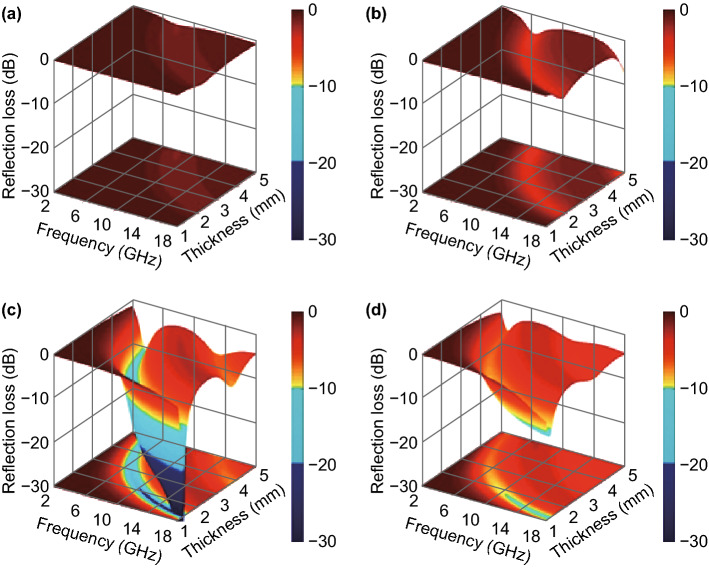
Three-dimensional RL maps of **a** WCC-2, **b** WCC-4, **c** WCC-6, and **d** WCC-8

Based on transmission line theory [59], microwave absorption performance of tungsten carbide/carbon composites can be calculated with these measured EM parameters by Eqs. 3 and 4,

3 $$ {\text{RL}}\;({\text{dB}}) = 20\log \left| {\frac{{Z_{\text{in}} - 1}}{{Z_{\text{in}} + 1}}} \right| $$

4 $$ Z_{\text{in}} = \sqrt {\frac{{\mu_{r} }}{{\varepsilon_{r} }}} \tanh \left[ {j\left( {\frac{2\pi }{c}} \right)fd\sqrt {\mu_{\text{r}} \cdot \varepsilon_{\text{r}} } } \right] $$

where *c*, *f*, and *d* correspond the velocity of EM wave in free space (i.e., 3 × 10^8^ m/s), the frequency of EM wave, and the thickness of MAMs, respectively. Figure 6 plots the three-dimensional RL maps of four tungsten carbide/carbon composites by employing *f* (2.0–18.0 GHz) and *d* (1.0–5.0 mm) as two independent variables. As observed, WCC-2 fails to produce any effective microwave absorption (Fig. 6a) due to its feeble dielectric loss ability and inactive magnetic loss ability (Figs. 5 and S5). Although the increase in dielectric loss ability brings more or less improvement in the performance of WCC-4 (Fig. 6b), its minimum RL intensity is still larger than − 10 dB, a commonly indicative value for qualified microwave absorption that equals to 90% absorption efficiency [47, 59]. In contrast, WCC-6 displays much better microwave absorption performance (Fig. 6c), whose strongest RL intensity can reach up to − 55.6 dB (*d *= 1.34 mm, *f *= 17.5 GHz) and qualified absorption bandwidth covers the frequency range of 3.6–18.0 GHz. The cutoff of RL value in Fig. 6c is artificially set at − 30.0 dB for a clear comparison, while the corresponding RL curve with the strongest absorption can be identified in Fig. S8. With further increasing carbon content, WCC-8 does not promise an expected enhancement in microwave absorption (Fig. 6d), and instead, its strongest RL intensity and qualified absorption bandwidth fall back to − 11.3 dB (*d *= 1.20 mm, *f *= 18.0 GHz) and 7.0 GHz (11.0–18.0 GHz). It is undoubted that WCC-6 is the best candidate among these tungsten carbide/carbon composites, no matter from RL intensity or qualified absorption bandwidth. In order to validate the performance of WCC-6 with those reported carbide/carbon composites, we summarize their RL characteristics in some appointed frequency intervals (Table 1) [28–31, 34–37, 60–63]. In general, researchers pay more attention to qualified absorption bandwidth, because there is no essential difference when RL intensity exceeds − 10.0 dB. One can easily find that WCC-6 indeed possess top-level microwave absorption performance in different frequency ranges, and it can even realize equivalent bandwidth with less *d* value. Simple synthesis and good performance offer a good platform for the practical application of such tungsten carbide/carbon composites.

**Table 1:** Microwave absorption performance of some typical carbide/carbon composites in previous references and this work

Absorbers	C band			X band			Ku band			References
	*d* (mm)	Min RL (dB)	Bandwidth (Range, GHz) (RL < − 10 dB)	*d* (mm)	Min RL (dB)	Bandwidth (Range, GHz) (RL < − 10 dB)	*d* (mm)	Min RL (dB)	Bandwidth (Range, GHz) (RL < − 10 dB)	
SiC/carbon fibers	3.00	− 33.5	1.7 (5.3–7.0)	2.00	− 23.0	2.4 (8.5–10.9)	1.50	− 19.0	2.3 (12.0–14.3)	[28]
Ti_3_C_2_Tx/CNTs	2.65	− 52.5	1.7 (6.3–8.0)	2.20	− 53.0	2.7 (8.2–10.9)	1.55	− 29.2	4.5 (13.5–18.0)	[29]
SiC/carbon foam	2.50	− 17.8	1.1 (5.6–6.7)	1.75	− 17.2	1.3 (8.3–9.6)	1.00	− 27.6	2.9 (15.0–17.9)	[30]
SiC@C nanocable	4.08	− 40.0	2.3 (5.3–7.6)	3.08	− 30.0	3.0 (8.0–11.0)	2.08	− 51.5	5.2 (12.8–18.0)	[31]
Mo_2_C/carbon	2.75	− 15.2	0.2 (7.8–8.0)	2.60	− 49.0	3.2 (8.0–11.2)	1.85	− 23.5	4.3 (12.0–16.3)	[34]
Mo_2_C@C nanorods	3.00	− 31.3	2.0 (5.7–7.7)	2.00	− 39.2	2.7 (9.3–12.0)	2.00	− 9.1	0	[35]
Mo_2_C@C nanospheres	3.50	− 18.9	2.2 (5.3–7.5)	2.50	− 26.7	3.0 (8.0–11.0)	1.50	− 32.1	3.5 (14.5–18.0)	[36]
Mo_2_C/C polyhedrons	3.50	− 27.3	1.6 (5.2–6.8)	2.00	− 40.9	2.3 (9.7–12.0)	1.50	− 60.4	4.8 (13.2–18.0)	[37]
Ti_3_C_2_/N-GP	4.00	− 35.0	1.9 (5.2–7.1)	2.50	− 30.2	3.2 (8.8–12.0)	1.50	− 22.5	3.0 (15.2–18.0)	[60]
SiC/graphene aerogel	3.50	− 34.6	1.7 (4.9–6.6)	2.50	− 16.3	4.0 (8.0–12.0)	1.50	− 21.7	3.2 (14.3–17.5)	[61]
B_4_C/amorphous carbon	2.70	− 24.6	0.5 (7.2–7.7)	2.00	− 43.4	1.0 (10.3–11.3)	1.50	− 60.9	1.5 (14.6–16.1)	[62]
Ti_3_C_2_Tx@RGO	4.00	− 27.5	2.0 (5.0–7.0)	2.50	− 26.9	3.3 (8.7–12.0)	2.00	− 25.8	4.7 (12.0–16.7)	[63]
WCC-6	3.03	− 15.0	2.0 (6.0–8.0)	2.13	− 20.5	3.2 (8.8–12.0)	1.50	− 26.3	4.9 (13.1–18.0)	Herein

It has to mention that there is a common relationship between the thickness and the frequency corresponding to minimum RL peaks in all these composites, i.e., a large *d* value can induce the shift of absorption peak to low-frequency region (Fig. 6). This phenomenon implies that incident EM waves may be attenuated based on a quarter-wavelength (*λ*/4) matching model. If this model works, the incident EM waves and reflected waves from a metal-backed layer with a phase difference at 180° will offset each other through destructive interference and realize the consumption of EM energy. In that case, the thickness of MAMs and the frequency of minimum absorption peak may satisfy Eq. 5 [33, 64]:

5 $$ t_{\text{m}} = \frac{n}{4}\lambda_{\text{m}} = \frac{nc}{{4f_{\text{m}} \sqrt {\left| {\varepsilon_{\text{r}} \cdot \mu_{\text{r}} } \right|} }}(n = 1,3,5 \ldots ) $$

where *t*_m_ and *f*_m_ are theoretical thickness and frequency, respectively. As shown in Fig. S9, the specific *d* values (green stars) of these tungsten carbide/carbon composites are exactly dispersed on the curves of *t*_m_ versus *f*_m_ (orange lines), confirming that EM dissipation in these composites is in good agreement with the quarter-wavelength matching model, that is, a strong EM extinction effect will occur at the specific values of *d* and *f*.

**Fig. 7:**
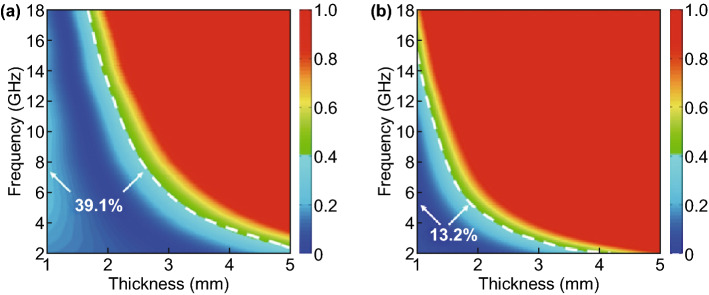
Delta maps of **a** WCC-6 and **b** WCC-8 with different absorber thickness in the frequency range of 2.0–18.0 GHz

It is widely accepted that RL characteristics of MAMs are highly dependent on their overall attenuation ability from both dielectric loss and magnetic loss [59, 65]. In our case, magnetic components are excluded in all these tungsten carbide/carbon composites, and thus, the attenuation constant (*α*) is determined by their dielectric loss alone. As confirmed in Fig. S10, all these composites give monotonously increased *α* values in the frequency range of 2.0–18.0 GHz, and the order of *α* value at a specific point is exactly consistent with that of dielectric loss tangent (Fig. S6). However, WCC-8 with the largest *α* value is incapable of generating the best microwave absorption performance (Fig. 6). This is because RL characteristics are also related to another important factor, impedance matching degree [66]. If the impedance of MAMs is poorly matched with that of free space, a strong reflection of incident EM waves will occur at the interface. As a result, no matter how strong their intrinsic attenuation ability is, they will not bring desirable microwave absorption performance. The ideal condition for perfect impedance matching requires that MAMs have identical *ε*_r_ and *μ*_r_, while it is impossible except in a wave-transparent medium that has negligible dielectric loss and magnetic loss [67, 68]. In common media dominated by dielectric loss, *ε*_r_ is usually much larger than *μ*_r_, which means that the gap between *ε*_r_ and *μ*_r_ should be tailored within a rational range to fulfill good attenuation ability and impedance matching simultaneously [37, 69]. We further employ a delta-function method to visibly illustrate the difference of impedance matching degree in WCC-6 and WCC-8 (Fig. 7). In this figure, the modules of delta values at different frequency points and absorber thickness can be calculated by Eqs. 6–8 [70],

6 $$ \left| \Delta \right| = \left| {\sinh^{2} \left( {Kfd} \right) - M} \right| $$

7 $$ K = \frac{{4\pi \sqrt {\mu_{\text{r}}^{{\prime }} \cdot \varepsilon_{\text{r}}^{{\prime }} } \cdot \sin \frac{{\delta_{\text{e}} + \delta_{\text{m}} }}{2}}}{{c \cdot \cos \delta_{\text{e}} \cdot \cos \delta_{\text{m}} }} $$

8 $$ M = \frac{{4\mu_{\text{r}}^{{\prime }} \cdot \cos \delta_{\text{e}} \cdot \varepsilon_{\text{r}}^{{\prime }} \cdot \cos \delta_{\text{m}} }}{{\left( {\mu_{\text{r}}^{{\prime }} \cdot \cos \delta_{\text{e}} - \varepsilon_{\text{r}}^{{\prime }} \cdot \cos \delta_{\text{m}} } \right) + \left[ {\tan \left( {\frac{{\delta_{\text{m}} }}{2} - \frac{{\delta_{\text{e}} }}{2}} \right)} \right]^{2} \cdot \left( {\mu_{\text{r}}^{{\prime }} \cdot \cos \delta_{\text{e}} + \varepsilon_{\text{r}}^{{\prime }} \cdot \cos \delta_{\text{m}} } \right)^{2} }}. $$

The smaller the modules of delta values are, the better impedance matching degree is achieved, and the threshold for good impedance matching is usually accepted as $\left| \Delta \right| \le 0.4$ [69, 70]. It is clear that WCC-6 possesses a good matching region with the coverage ratio at about 39.1%, while the coverage of matching region in WCC-8 drastically shrinks back to 13.2%. These results explain that the recession of microwave absorption performance in WCC-8 is mainly attributed to its deteriorative impedance matching degree. To support this viewpoint, we further raise the DCA/AM weight ratio to 10.0, and the relative complex permittivity and dielectric loss tangent of WCC-10 are further enhanced as compared with WCC-8 (Fig. S11a, b). It is unfortunate that the impedance matching degree is further deteriorated, whose coverage of matching region is only 3.1% (Fig. S11c), again verifying that the overlarge gap between relative complex permittivity and complex permeability is not beneficial to good impedance matching. Correspondingly, WCC-10 cannot produce good microwave absorption performance due to strong reflection induced by such poor impedance matching (Fig. S11d). That is to say, the pursuit of strong dielectric loss ability is not certainly favorable for RL characteristics, and the impedance matching should be also taken into account seriously.

## Conclusions

Tungsten carbide/carbon composites have been successfully prepared with the solid mixture of dicyandiamide (DCA) and ammonium metatungstate (AM) as a precursor. Ultrafine cubic tungsten carbide nanoparticles in situ formed during high-temperature pyrolysis are homogeneously dispersed on carbon nanosheets. It is found that the weight ratio of DCA to AM plays an important role in determining the relative contents of carbon nanosheets and WC_1-*x*_ nanoparticles, as well as their dielectric properties. The composite WCC-6 (DCA:AM = 6:1) exhibits good microwave absorption, whose strongest reflection loss reaches up to − 55.6 dB and qualified absorption bandwidth covers the frequency range of 3.6–18.0 GHz. Such a performance benefits from its decent attenuation ability and desirable impedance matching and is indeed superior to many conventional carbides/carbon composites. It is believable that these results are not only helpful for the preparation of high-performance microwave-absorbing materials in a green and sustainable way, but also inspire the development of functional carbide/carbon composites in other fields.

## Electronic Supplementary Material

Below is the link to the electronic supplementary material.

Supplementary material 1 (PDF 935 kb)
