# Supplementary material for: Solvent-Free Synthesis of Ultrafine Tungsten Carbide Nanoparticles-Decorated Carbon Nanosheets for Microwave Absorption
Source: Nanomicro Lett. 2020 Jul 18;12:153. doi: 10.1007/s40820-020-00491-5 (PMC7770940; doi:10.1007/s40820-020-00491-5)
Supplement: Supplementary file 1 — Supplementary material 1 (PDF 935 kb) [file 40820_2020_491_MOESM1_ESM.pdf]

Supporting Information for

Solvent-Free Synthesis of Ultrafine Tungsten Carbide Nanoparticles Decorated Carbon Nanosheets for Microwave Absorption

Yunlong Lian[1], Binhua Han[1], Dawei Liu[1], Yahui Wang[1], Honghong Zhao[1], Ping Xu[1], Xijiang Han[1,*], Yunchen Du[1,*]

Yunlong Lian and Binhua Han contributed equally to this work.

[1]MIIT Key Laboratory of Critical Materials Technology for New Energy Conversion and Storage, School of Chemistry and Chemical Engineering, Harbin Institute of Technology, Harbin 150001, People's Republic of China

*Corresponding authors. E-mail: yunchendu@hit.edu.cn (Yunchen Du); hanxijiang@hit.edu.cn (Xijiang Han)

Supplementary Figures

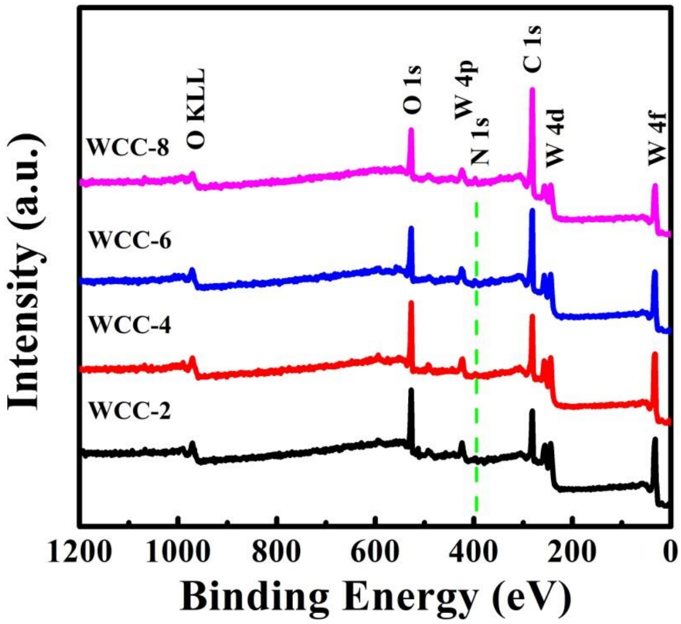

Fig. S1 Survey XPS spectra of different tungsten carbide/carbon composites

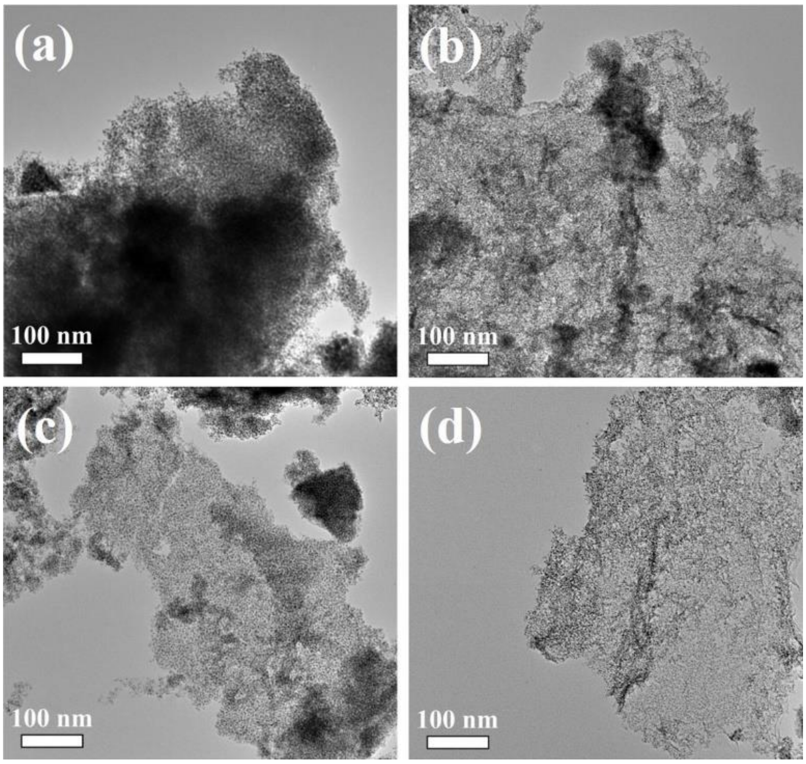

Fig. S2 Low-magnification TEM images of **a** WCC-2, **b** WCC-4, **c** WCC-6, and **d** WCC-8

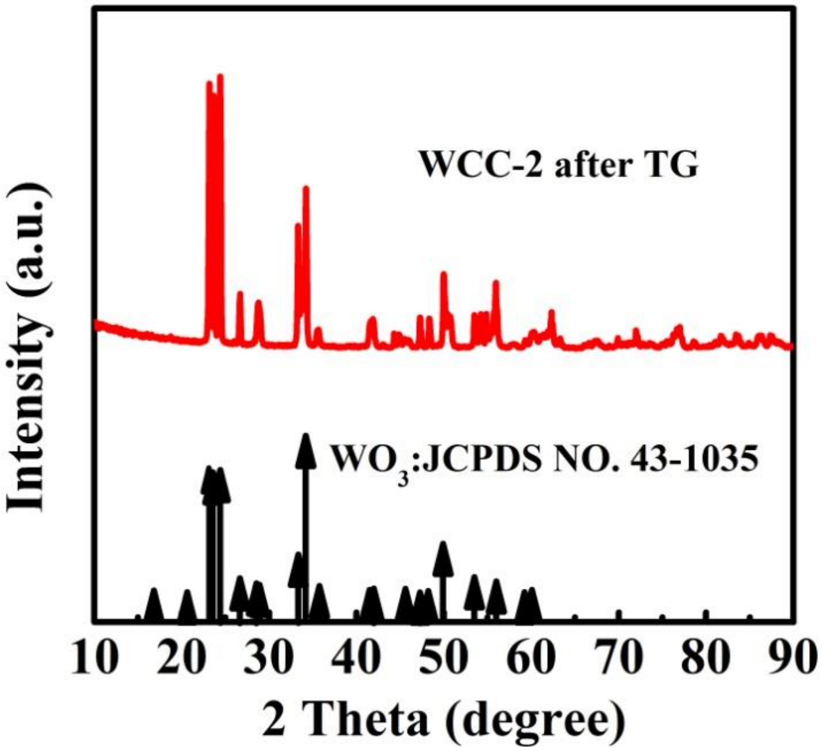

Fig. S3 XRD pattern of the final product of WCC-2 after TG measurement

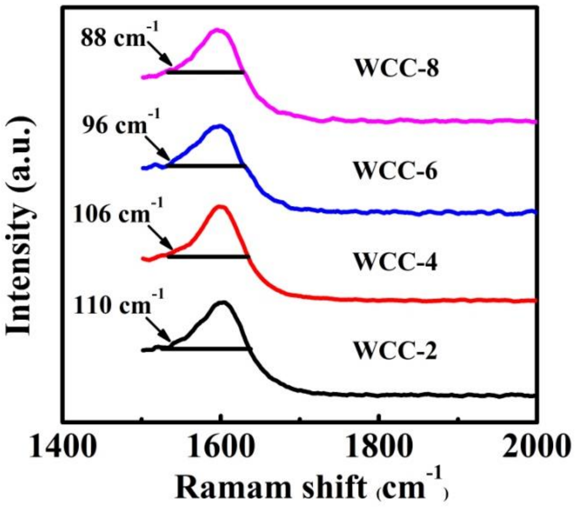

Fig. S4 The local amplification of G band in Raman spectra of different tungsten carbide/carbon composites

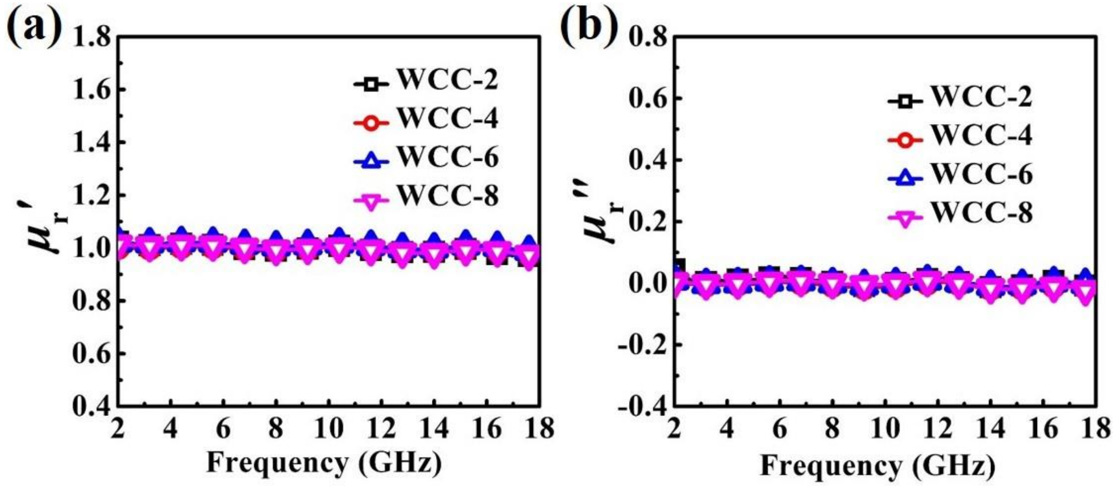

Fig. S5 a Real parts and **b** imaginary parts of relative complex permeability of different tungsten carbide/carbon composites

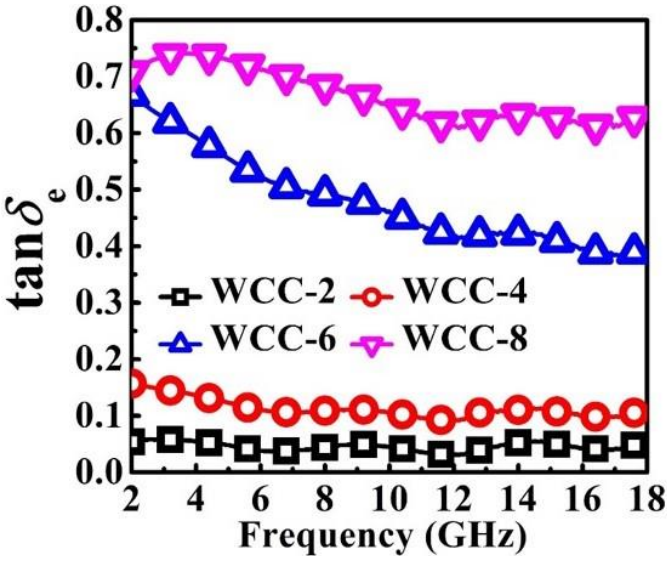

Fig. S6 Dielectric loss tangents of different tungsten carbide/carbon composites

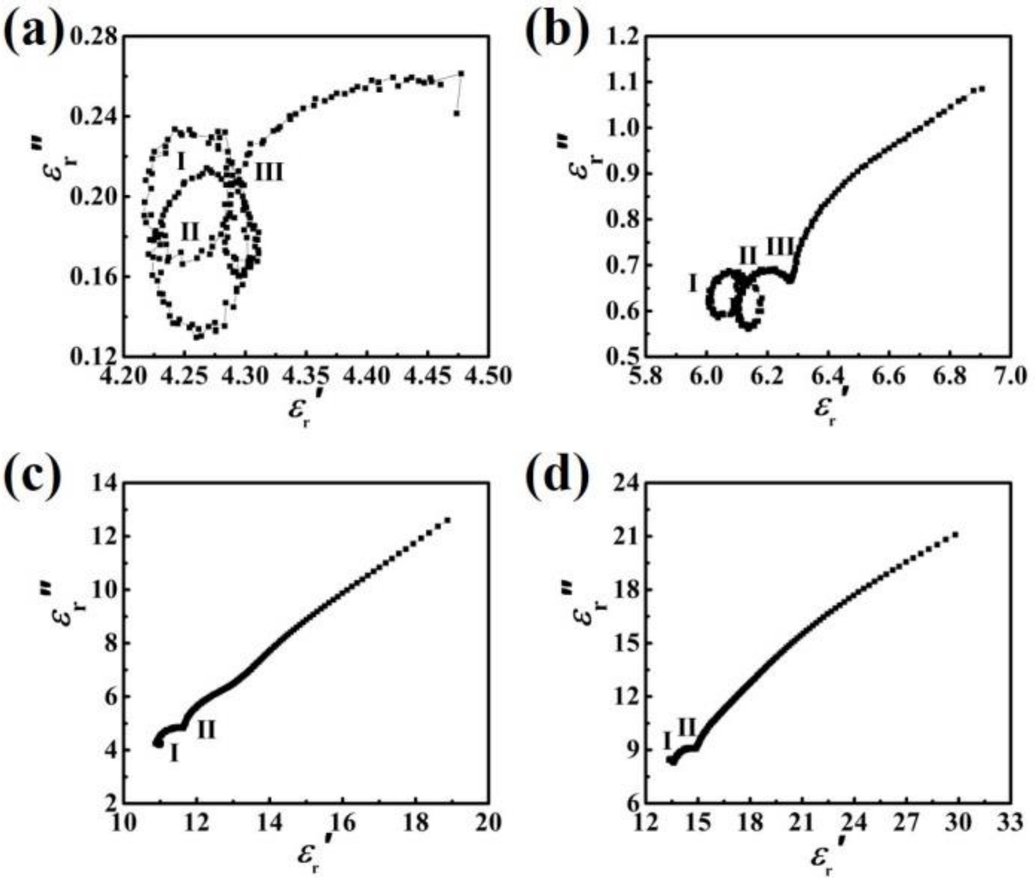

Fig. S7 The curves of ε_r'' *vs.* ε_r' (Cole–Cole semicircles) of **a** WCC-2, **b** WCC-4, **c** WCC-6, and **d** WCC-8

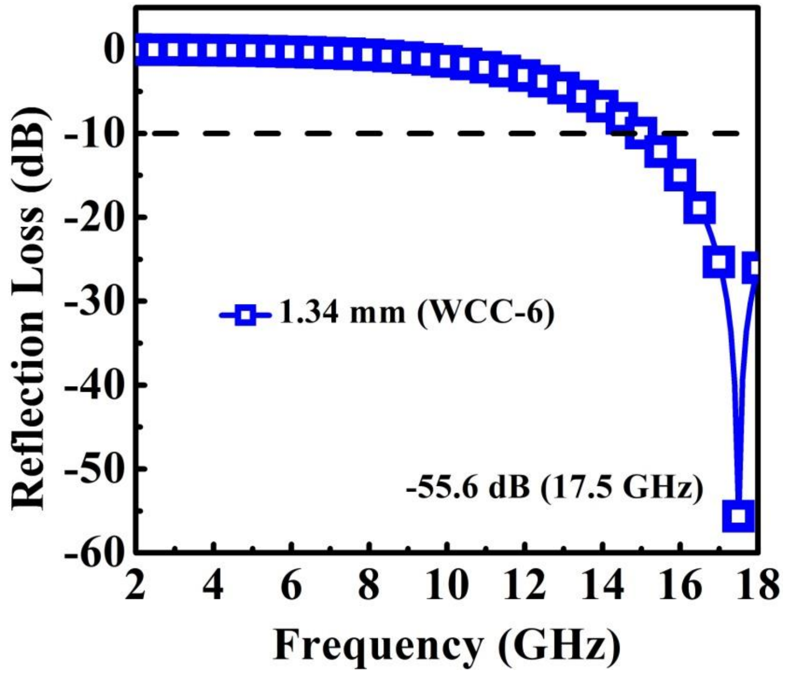

Fig. S8 RL curve of WCC-6 with the thickness of 1.34 mm

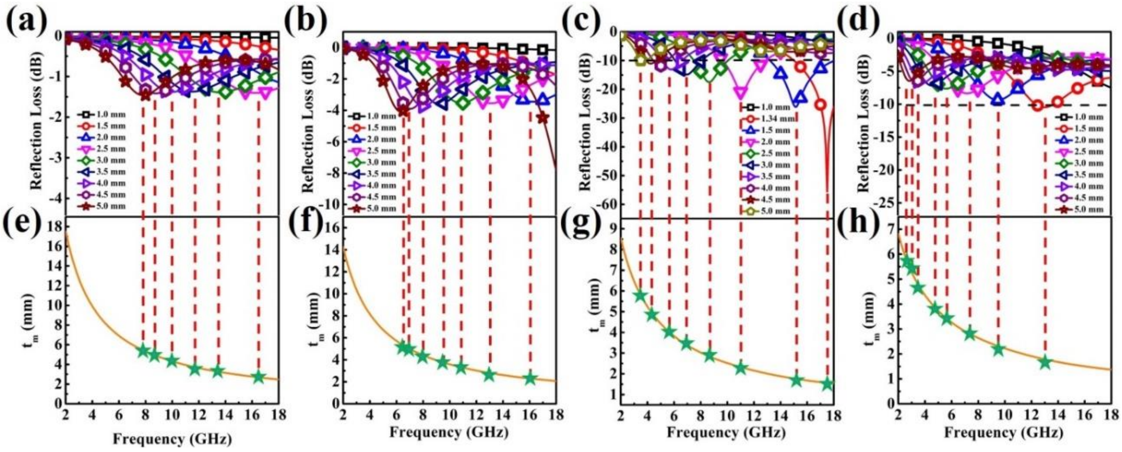

Fig. S9 RL curves and dependence of matching thickness (t_m) on matching frequency (f_m) of **a, e** WCC-2, **b, f** WCC-4, **c, g** WCC-6, and **d, h** WCC-8.

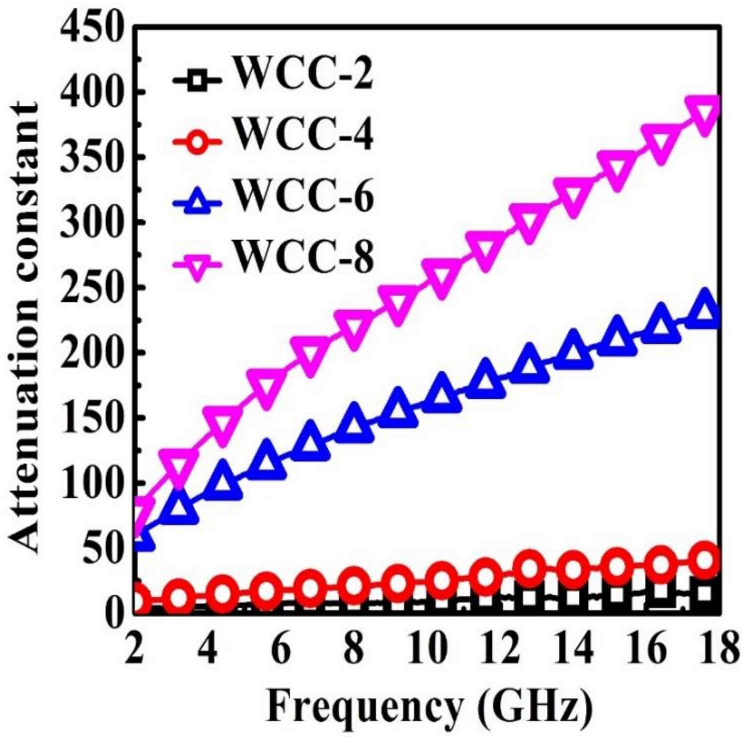

Fig. S10 Frequency-dependent attenuation constants (α) of different tungsten carbide/carbon composites

The values of α can be calculated by the following equation:

$$\alpha = \frac{\sqrt{2}\pi f}{c}\sqrt{(\mu_r''\varepsilon_r'' - \mu_r'\varepsilon_r') + \sqrt{(\mu_r''\varepsilon_r'' - \mu_r'\varepsilon_r')^2 + (\mu_r'\varepsilon_r'' + \mu_r''\varepsilon_r')^2}}$$

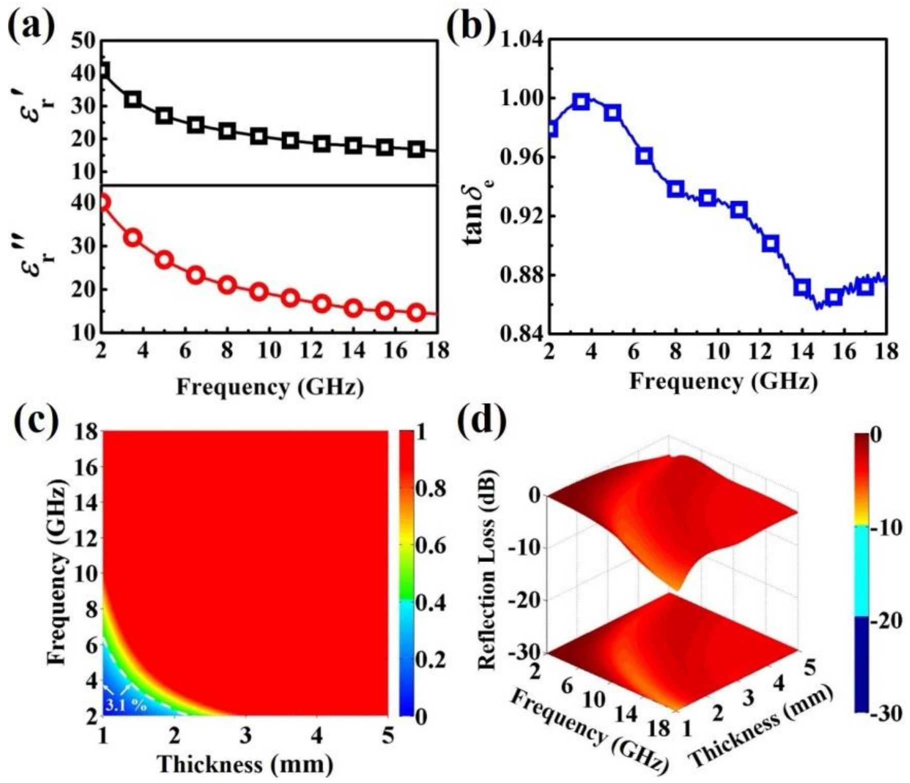

Fig. S11 a Relative complex permittivity, **b** dielectric loss tangent, **c** delta map, and **d** RL map of WCC-10
